# Rotavirus A genotype diversity and antigenic profile in Central Ethiopia: implications for rotarix^®^ vaccine efficacy

**DOI:** 10.3389/fmicb.2025.1656797

**Published:** 2025-10-07

**Authors:** Yisehak Tsegaye Redda, Haileeyesus Adamu, Julia Bergholm, Johanna F. Lindahl, Anne-Lie Blomström, Mikael Berg, Tesfaye Sisay Tessema

**Affiliations:** ^1^Biotechnology Research Center, Institute of Advanced Sciences and Technology, Addis Ababa University, Addis Ababa, Ethiopia; ^2^Department of Animal Biosciences, Swedish University of Agricultural Sciences, Uppsala, Sweden; ^3^Department of Veterinary Basic and Diagnostic Sciences, College of Veterinary Sciences, Mekelle University, Mekelle, Ethiopia; ^4^Department of Medical Biochemistry and Microbiology, Uppsala University, Uppsala, Sweden; ^5^Department of Animal Health and Antibiotic Strategies, Swedish Veterinary Agency, Uppsala, Sweden

**Keywords:** Central Ethiopia, gastroenteritis, rotavirus, molecular epidemiology, VP7 and VP4 genes, vaccine efficacy

## Abstract

**Introduction:**

Rotavirus remains a leading cause of severe gastroenteritis in children globally, including in Ethiopia. Despite the introduction of vaccines, high mutation and reassortment rates contribute to genetic diversity and potential vaccine escape. This study aimed to assess the distribution and genetic characteristics of rotavirus A (RVA) strains in children under five with diarrhea in central Ethiopia, with comparison to the Rotarix^®^ vaccine strain.

**Methods:**

Stool samples were collected from children under 5 years of age presenting with diarrhea at health centers in Debre Berhan and Addis Ababa between April 2022 and December 2023. RVA was detected using quantitative real-time PCR (qPCR). Genotyping was performed by Sanger sequencing of the VP7 and VP4 genes. Phylogenetic analysis was performed in MEGA X software using the maximum likelihood method with 1,000 bootstrap replicates, using reference sequences retrieved from the GenBank database. Amino acid sequences of these proteins were compared with those of the Rotarix^®^ vaccine strain to identify substitutions in key antigenic regions.

**Results:**

RVA was detected in 30 of 247 samples (12.14%), with 28 successfully genotyped. G9 was the predominant G genotype (50%), followed by G12 (10.2%), G2 (7.1%), G1 (3.6%), and G3 (3.6%); 25% remained untyped. P[4] was the most common P genotype (28.6%), followed by P[6] (21.4%) and P[8] (17.9%), with 32.1% untyped. The most frequent G/P combinations were G9P[4] (35%), G12P[6] (13%), and G9P[8] (9%). Compared to Rotarix^®^, the circulating G2, G3, G9, and G12 strains showed 18, 12, 13, and 17 amino acid substitutions, respectively, within the 29-residue VP7 epitopes. The P[8], P[4], and P[6] strains exhibited 4, 9, and 18 substitutions, respectively, within the 28 VP4 neutralizing epitope residues. Phylogenetic analysis revealed that the current identified virus mainly clusters with strains previously reported from Ethiopia, indicating a shared evolutionary origin.

**Conclusion:**

The dominance of the G9P[4] genotype, together with substantial amino acid substitutions in the current circulating RVA strains that diverge from the G1P[8] Rotarix^®^ strain, may compromise vaccine performance. These findings underscore the need to evaluate vaccine efficacy, maintain molecular surveillance, and incorporate broader genotype coverage in future vaccine design.

## Introduction

Diarrheal diseases remain one of the leading causes of childhood mortality globally, second only to respiratory tract infections, with an estimated 1.7 billion cases and over 500,000 deaths annually in children under 5 years of age (Hodges and Kelly, 2023). Among diarrheal pathogens, rotavirus is the leading cause of severe gastroenteritis in young children worldwide (Chen et al., 2012).

In 2016, the World Health Organization (WHO) estimated that rotavirus was responsible for approximately 215,000 deaths in children under 5 years of age, with Sub-Saharan Africa accounting for 121,000 (56.3%) of these deaths. In Ethiopia alone, 6,817 rotavirus-related deaths were reported in the same year (Tate et al., 2016).

The prevention of rotavirus gastroenteritis, particularly in children, is significantly enhanced by the use of rotavirus vaccines. WHO's routine-infant vaccination recommendation for Rotarix^®^ and RotaTeq^®^ has driven substantial global declines in rotavirus cases, hospitalizations, and deaths (Mwenda et al., 2010).

Rotarix^®^ has demonstrated substantial impact on rotavirus disease burden, reducing infection rates from 36% to 22%, which corresponds to an overall effectiveness of 69% (Kazimbaya et al., 2018; Willame et al., 2018). However, its performance varies significantly by region. In high-income countries, vaccine effectiveness reaches approximately 81%, whereas in low-and middle-income countries, it averages around 53%. Within Africa, pooled analyses indicate a 58 % effectiveness against rotavirus-associated hospitalizations among children who completed the two-dose schedule, compared to 44% for those receiving only a single dose (Murunga et al., 2020).

Rotaviruses are non-enveloped, triple-layered viruses belonging to the *Sedoreoviridae* family. Their genome consists of 11 segments of double-stranded RNA (dsRNA) encoding six structural proteins (VP1–VP4, VP6, and VP7) and six non-structural proteins (NSP1–NSP6) (Pesavento et al., 2006). Group A rotaviruses (RVA) are the primary cause of acute gastroenteritis in humans and are classified into G and P genotypes based on the VP7 and VP4 gene segments, respectively. Globally, five G types (G1–G4 and G9) and three P types (P[4], P[6], and P[8]) account for over 90% of human RVA infections (Miranda et al., 2024; Santos and Hoshino, 2005). However, rotavirus genotypes show marked spatiotemporal variation, and the emergence of novel or unusual strains is driven by reassortment, recombination, and point mutations (Tcheremenskaia et al., 2007; Matthijnssens et al., 2011).

In Ethiopia, hospital-based studies have consistently identified RVA as the leading cause of non-bacterial acute gastroenteritis in infants and young children, accounting for 18–28% of cases (Sahiledengle et al., 2024; Tosisa et al., 2024). Furthermore, Meta-analysis data have shown evidence of a shift in predominant genotypes after vaccine introduction (Tosisa et al., 2024).

Despite the 2013 introduction of the monovalent Rotarix^®^ vaccine (G1P[8]) into Ethiopia's national immunization program and a reported 69.8% coverage (Aliyo, 2022), recent data on circulating RVA genotypes, vaccine effectiveness, and the emergence of potential vaccine breakthrough genotypes remain limited.

Given the dynamic nature of RVA epidemiology and the variability in genotype distribution across geographic regions and over time, continuous molecular surveillance is critical. Understanding the current genotypic landscape is essential for detecting emergent strains and guiding future immunization strategies.

This study aimed to characterize the distribution and genetic diversity of RVA strains in diarrheic children in central Ethiopia and assess their genetic similarity and antigenic divergence from the Rotarix^®^ vaccine strain.

## Methods

### Study design and sample collection

A cross-sectional study was conducted between April 2022 to December 2023 from Debrebirhan City health post in Debrebirhan and Nifasilk lafto sub-city health post in Addis Ababa, central Ethiopia. A total of 247 fecal samples were collected from children under 5 years of age who had diarrhea and visited health service centers as outpatients in the study area. The study included children who presented with a passage of at least three watery or liquid stools per day for less than 14 days, with or without vomiting. The samples were collected as part of the routine diagnostic procedure at the laboratory.

The samples were collected in sterile stool cups and transported under a cold chain to the Institute of Biotechnology, Addis Ababa University (AAU). A 10% fecal suspension was prepared with phosphate-buffered saline. The mixture was vortexed vigorously and then centrifuged at 10,000 rpm for 5 min. The supernatant was transferred to new tubes and stored at−20 °C until RNA extraction.

### Detection and genetic characterization of RVA

#### RNA extraction and quality assessment

For RNA extraction, 250 μL of supernatant was mixed with 750 μL TRIzol reagent (Invitrogen), incubated for 5 min, and 150 μL chloroform was added and vortexed. After centrifugation at 12,000 rpm for 15 min at 4 °C, the upper aqueous phase was transferred to new tube, mixed with an equal volume of 70% ethanol, and purified using a GeneJET RNA purification kit (Thermo Fisher Scientific). RNA integrity was assessed using the TapeStation system (Agilent), following the manufacturer's instructions. The extracted RNA was stored at−80 °C.

#### RT-qPCR detection of RVA

RVA detection was performed using a qPCR targeting the NSP5 gene, as previously described by Bergholm et al. (2024). Briefly, reactions were carried out in a final volume of 30 μL using the 4 × TaqMan Fast Virus 1-Step Master Mix (Thermo Fisher Scientific). Each reaction contained 1 × master mix, 600 nM of each primer (forward: TGATTCTGCTTCAAACGATCCA; reverse: GCATTTGTCTTAACTGCATTCGA), 150 nM of TaqMan probe (VIC-TCACCAGCTTTTCGATAAG-MGB), 2 μL of RNA template, and nuclease-free water to volume. Positive and negative controls were included in each run. Amplification was performed on a CF X 96 Real-Time PCR Detection System (Bio-Rad) under the following cycling conditions: reverse transcription at 50 °C for 5 min, initial denaturation at 95 °C for 20 s, followed by 45 cycles of denaturation at 95 °C for 15 s and annealing/extension at 60 °C for 1 min, with fluorescence acquisition at each cycle.

#### cDNA synthesis

Complementary DNA (cDNA) was synthesized from qPCR-positive samples using the SuperScript IV cDNA Synthesis Kit (Thermo Fisher Scientific), following the manufacturer's protocol. Briefly, 5 μL of RNA template was combined with 1 μL of 50 ng/μL random hexamers and 1 μL of 10 mM dNTP mix in a total volume of 13 μL. The mixture was incubated at 95 °C for 5 min and then chilled on ice for 1 min. Subsequently, 4 μL of 5 × SuperScript IV buffer, 1 μL of 100 mM DTT, 1 μL of RNaseOUT™, and 1 μL of SuperScript IV Reverse Transcriptase were added, bringing the final volume to 20 μL. Reverse transcription was carried out at 50 °C for 10 min, followed by enzyme inactivation at 80 °C for 10 min. The resulting cDNA was stored at −20 °C until further use.

#### VP7 and VP4 gene amplification

For the VP7 gene, primers VP7F (5′-ATGTATGGTATTGAATATACCAC-3′) and VP7R (5′-AACTTGCCACCATTTTTTCC-3′) were used to amplify an 881 bp fragment. For the VP4 gene of the VP8^*^ region, primers con3 (5′-TGGCTTCGCTCATTTATAGACA-3′) and con2 (5′-ATTTCGGACCATTTATAACC-3′) were used to amplify an 877 bp fragment as previously described (World Health Organization, 2009). The PCR reactions were carried out using 2X Platinum SuperFi PCR Master Mix as per the manufacturer's instructions. Each 20 μL reaction mixture contained 1X of Platinum SuperFi PCR Master Mix (Invitrogen), 600 nM of both forward and reverse primers, and 2 μL of cDNA. The PCR cycling conditions were as follows: initial denaturation at 98 °C for 30 s, followed by 35 cycles of 98 °C for 10 s, 60 °C for 30 s, and 72 °C for 30 s, with a final extension at 72 °C for 5 min. The PCR products were subsequently separated on a 1% agarose gel, stained with GelRed™, and visualized using the ChemiDoc™ MP Imaging System.

#### Sequence analysis and genotype determination

PCR products were assessed for quality and specificity by agarose gel electrophoresis; sharp, single bands of expected size with minimal background were selected for sequencing. PCR products were purified using the GeneJET Gel Extraction Kit (Thermo Fisher Scientific) and Sanger sequenced (Macrogen Europe). Consensus sequences were assembled using Geneious Prime (v.2024.0.7). Genotype determination was initially performed by comparing the consensus sequences to reference nucleotide sequences using BLASTn (National Center for Biotechnology Information (NCBI), 2025). Rotavirus genotypes were further confirmed using the ViPR viral species identification tool (Pickett et al., 2012) and validated through phylogenetic analysis. The VP7 and VP4 gene sequences were submitted to GenBank under accession numbers PV009100–PV009139.

### Data analysis

RVA nucleotide sequences were aligned with global representative sequences retrieved from the GenBank database (National Center for Biotechnology Information, Bethesda, MD, USA) using ClustalW in MEGA X (Kumar et al., 2018). The best model for each dataset was determined using the “Find Best DNA/Protein model”, and maximum-likelihood phylogenetic trees were constructed using MEGA X software (Kumar et al., 2018). The statistical reliability was checked using 1,000 bootstrap replicates. Nucleotide and amino acid distances were calculated using the P Distance Model.

For antigenic characterization, deduced amino acid sequences of RVA were aligned with known epitope regions of the Rotarix^®^ vaccine strain using ClustalW, and alignments were visualized with the Jalview alignment tool (v2.11.3.3) to identify amino acid differences.

Structural analyses of VP7 (PDB ID: 3FMG) and the VP8^*^ domain of VP4 (PDB ID: 1KQR) were performed using the PyMOL Molecular Graphics System, Version 3.0 (Schrödinger, LLC). Major antigenic epitopes were annotated, including VP7 regions 7-1a, 7-1b, and 7-2, and VP8^*^ epitopes 8-1 to 8-4. Amino acid substitutions identified from sequence alignments were mapped onto the corresponding 3D structures. Comparative structural analysis was conducted between the circulating and vaccine strains.

## Results

A total of 247 children were enrolled in the study, with a mean age of 31.5 months. The majority of participants (57.5%) were between 13 and 36 months old, followed by 37–60 months (26.3%), and 0–12 months (7.7%). Most children (88.3%) resided in urban areas, and a slightly higher proportion were male (55.7%). By study site, the enrollment was distributed between Addis Ababa (56.3%) and Debre Birhan (43.7%) (Table 1). The overall occurrence of RVA infection among diarrheic children was 12.14% combining both study areas. The proportion was relatively equal in Addis Ababa 17/139 (12.2%), and Debre Birhan 13/108 (12.03%). Rotavirus infection showed a marginally significant association with age group (*p* = 0.08), with the highest infection rate observed among children aged 13–36 months (14.8%), followed by those aged 0–12 months (10.0%) and 37–60 months (7.7%). Infection was higher among rural residents (13.8%) compared to urban (11.9%) populations, although this difference was not statistically significant (*p* = 0.75). Male and female children had comparable infection rates (12.3% vs. 11.9%, *p* = 0.91).

**Table 1:** Sociodemographic characteristics of study participants (*n* = 247) and Rotavirus Prevalence.

**Characteristic**	**(*n*)(%)**	**Percentage (%)**	**Rotavirus cases (*n*)**	**Prevalence (%)**	***p-value***
**Age group (Months)**					
0–12	19	7.7	2	10.0	0.08
13–36	142	57.5	21	14.8	
37–60	65	26.3	5	7.7	
**Residence**					
Urban	218	88.3	26	11.9	0.75
Rural	29	11.7	4	13.8	
**Sex**					
Male	138	55.9	17	12.3	0.91
Female	109	44.1	13	11.9	
**Location**					
Addis Ababa	139	56.3	17	12.2	0.96
Debre Birhan	108	43.7	13	12.0	
**Overall**	247	100	30	12.1	—

**Figure 1:**
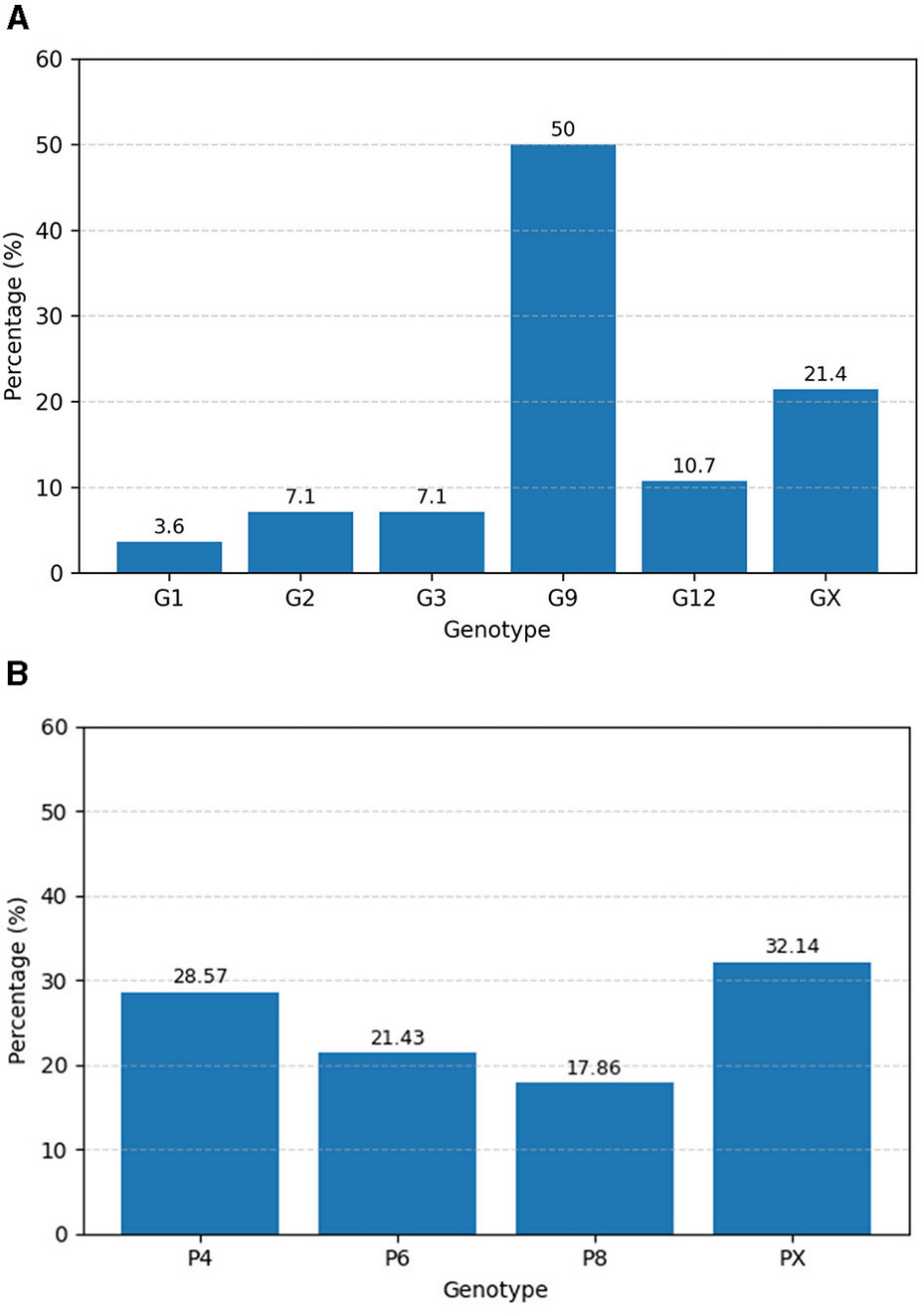
Percentage distribution of RVA G types **(A)** and P types **(B)** isolated from children with acute gastroenteritis in Addis Ababa and Debre Birhan, Central Ethiopia from April 2022–December 2023.

Out of 30 detected samples, 28 were used for genotyping. The identified circulating G-types included G1, G2, G3, G9, and G12, with G9 being the most prevalent genotype, detected in 50% (14/28) of the genotyped samples, followed by G12 at 10.7% (3/28) and G2 at 7.1% (2/28). The remaining 25% (7/28) of samples could not be G-typed (Figure 1A). Among the P-types, P[4], P[6], and P[8] were detected at proportions of 28.6% (8/28), 21.4% (6/28), and 17.9% (5/28), respectively, while 32.1% (9/28) of samples could not be classified into a P-type (Figure 1B). Various G/P genotype combinations were identified, with the most frequent being G9P[4] (35%), followed by G12P[6] (13%), G9P[8] (9%), G2P[6](4%), and G3P[8](4%) (Table 2).

**Table 2:** Percentage distribution of RVA G/P genotype combinations isolated from children with acute gastroenteritis in Addis Ababa and Debre Birhan, Central Ethiopia, from April 2022 to December 2023.

**G/P genotype combinations**	**Frequency**	**Percentage(%)**
G1PX	1	4.4
G2PX	1	4.4
G2P6	1	4.4
G3P8	1	4.4
G9P4	8	34.8
G9P8	2	8.7
G9PX	3	13.0
GXP8	2	8.7
GXP6	1	4.4
G12P6	3	13.0
Total	23	100

### Phylogenetic analysis of the VP7 gene of the circulating RVA strains

**Figure 2:**
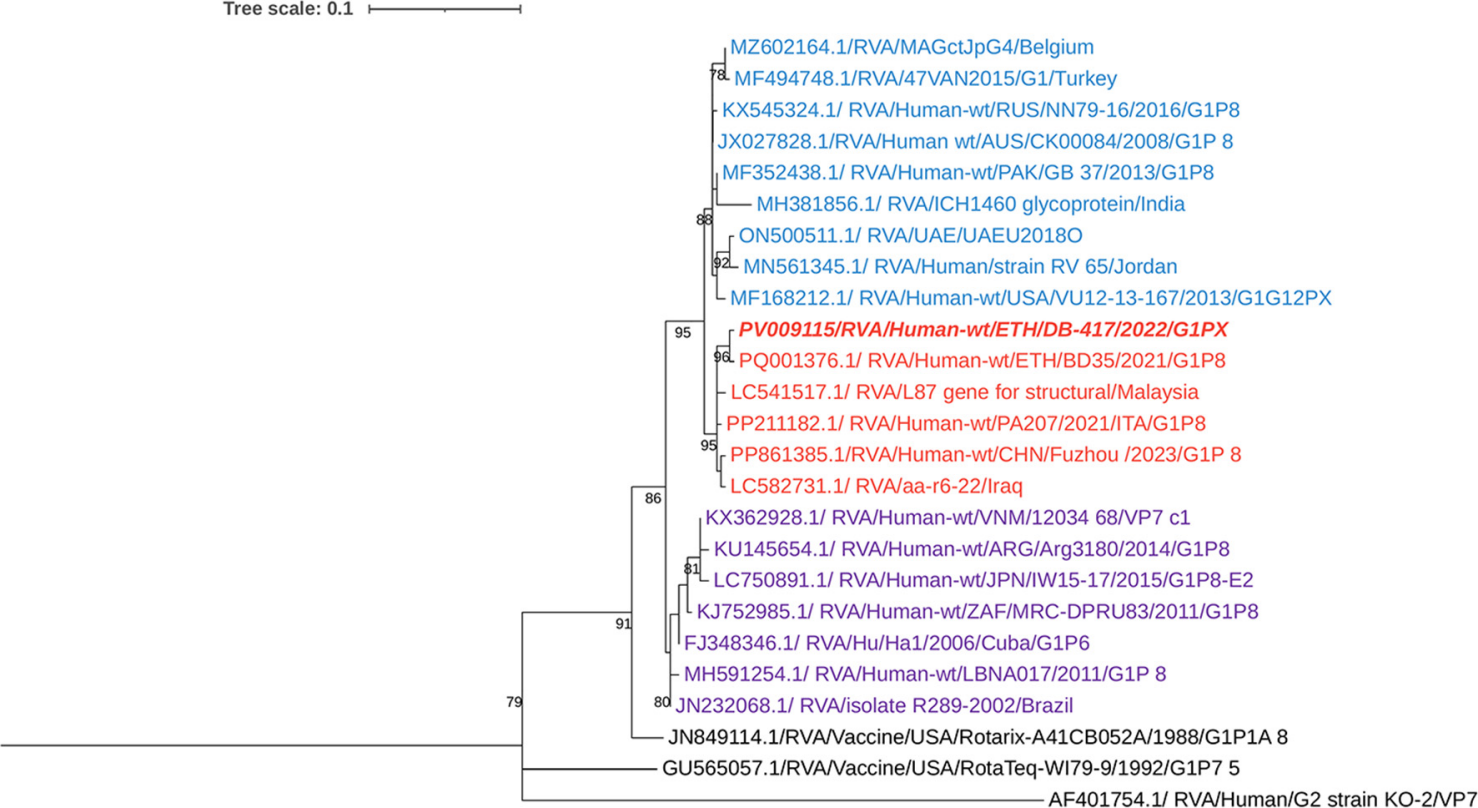
Maximum-likelihood trees of rotavirus G1 strains were constructed based on the partial VP7 CDS region sequences (881 base pairs). A T92 + G nucleotide substitution model was used to construct the phylogenetic tree. The human RVA G2 strain (AF401754.1) was used as the outgroup. The current Ethiopian strain are written in bold and italic. Each color represents a specific clade. Bootstrap values (1,000 replicates) of >70% are shown at the nodes.

Phylogenetic analysis demonstrated that the current RVA G1 isolates clustered closely with human G1 strains previously reported in Ethiopia, Malaysia, Italy, China, and Iran (Figure 2). In contrast, the Rotarix^®^ vaccine G1 strain formed a distinct phylogenetic cluster, separate from the group containing the Ethiopian isolates. The current G1 strain shared the highest nucleotide similarity (99.73%) with earlier Ethiopian G1 strains and exhibited substantial identity (96.34%) with the G1 strain used in the Rotarix^®^ vaccine.

**Figure 3:**
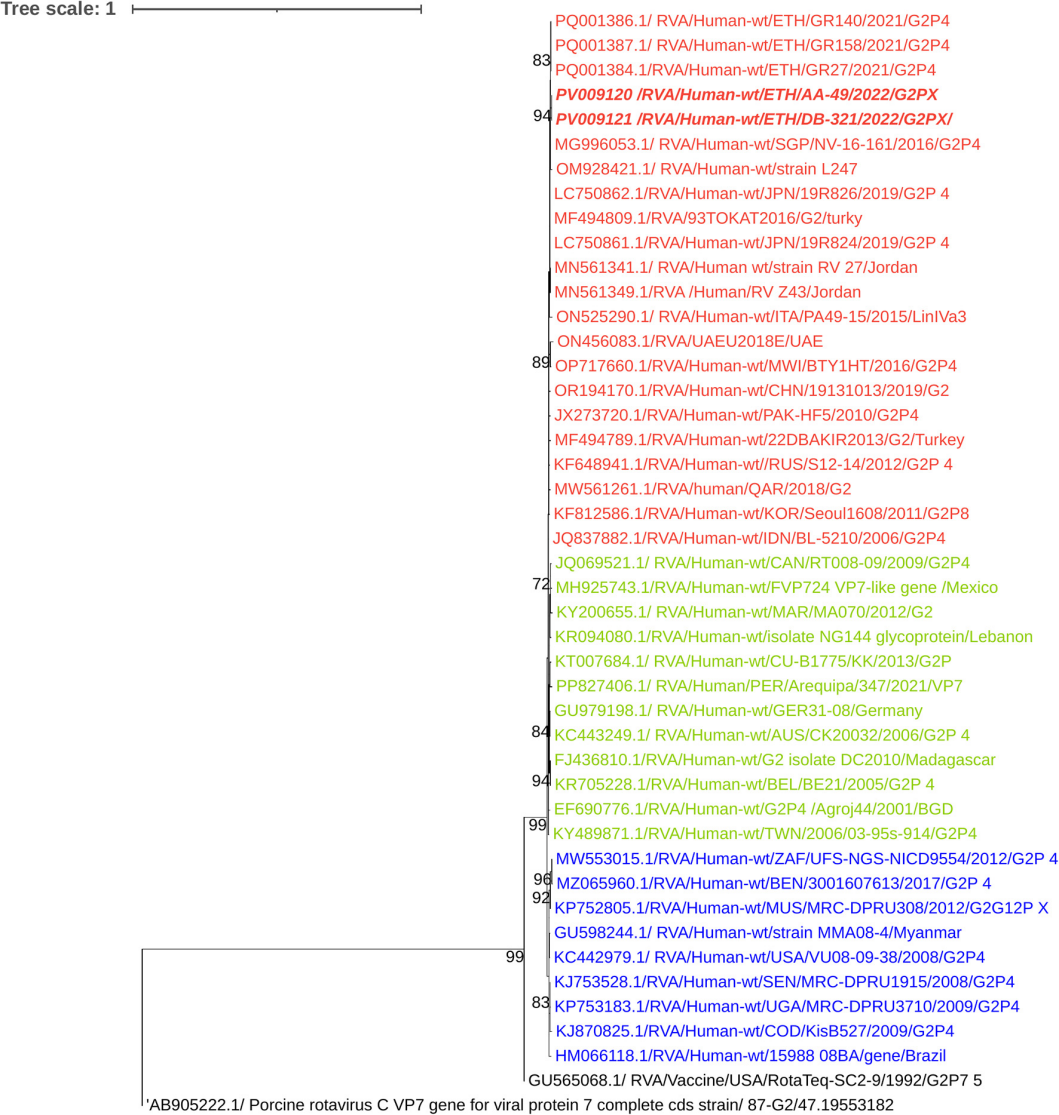
Maximum-likelihood trees of Rotavirus G2 Strains were constructed based on the partial VP7 CDS region sequences (881 base pairs). A T92 + G + I nucleotide substitution model was used to build the phylogenetic tree. Porcine Rotavirus Strain (AB905222.1.) was used as the outgroup. The current Ethiopian strains are written in bold and italic. Each color represents a specific clade. Bootstrap values (1,000 replicates) >70% are shown at the nodes.

The currently circulating G2 rotavirus strains (*n* = 2), collected from different locations, Addis Ababa and Debrebirhan, were found to be 100% identical to each other at the nucleotide level and exhibited 99.52% nucleotide similarity with previously reported Ethiopian G2 strains. Phylogenetic analysis revealed that the isolate clustered closely with human RVA G2 strains reported from Singapore, Japan, Turkey, Jordan, Italy, and Ethiopia (Figure 3).

**Figure 4:**
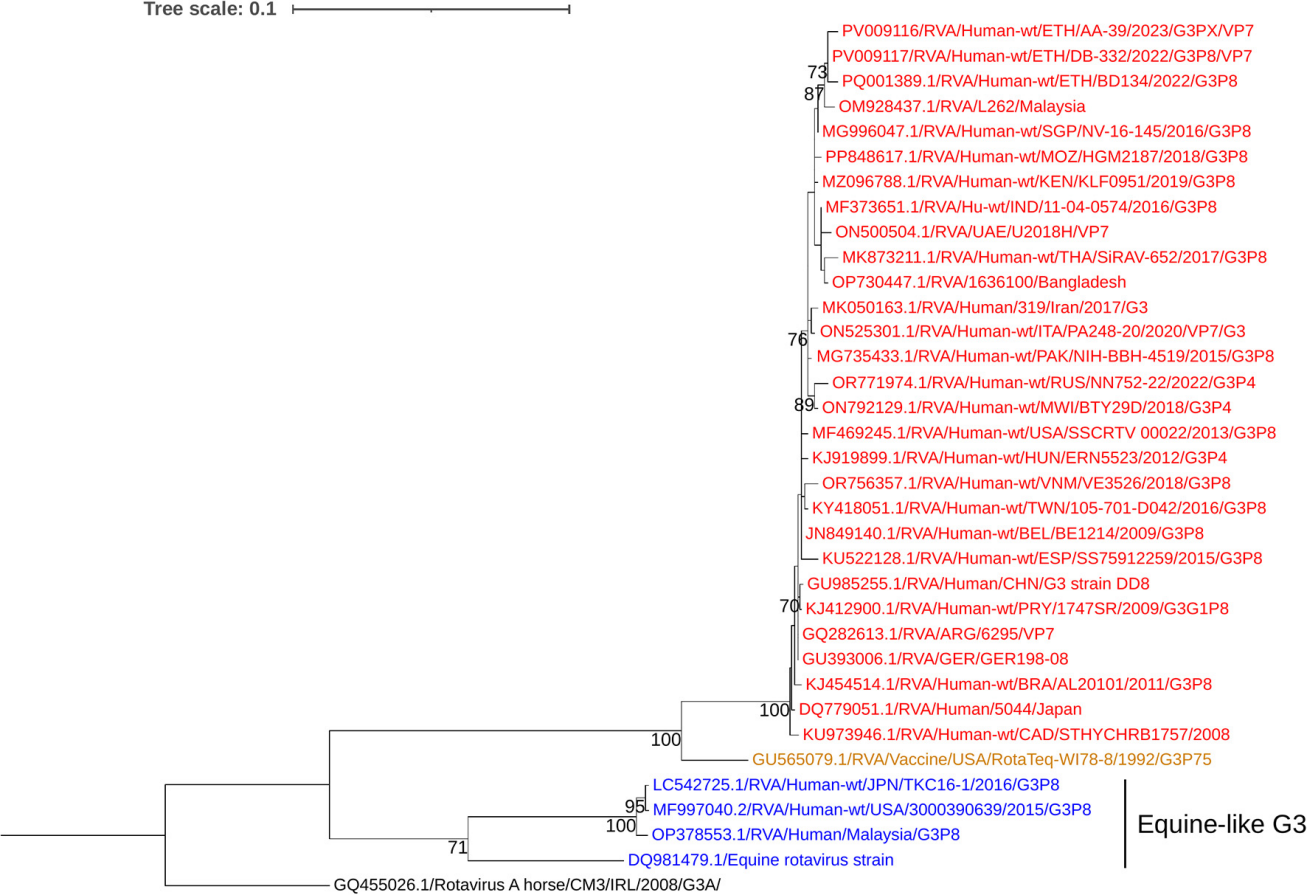
Maximum-likelihood trees of rotavirus G3 Strains were constructed based on the partial VP7 CDS region sequences (881 base pairs). A T92 + G nucleotide substitution model was used to construct the phylogenetic tree. The Equine RVA G3 strain (GQ455026.1) was used as the outgroup. The current Ethiopian strains are written in bold and italic. Each color represents a specific clade. Bootstrap values (1,000 replicates) >70% are shown at the nodes.

The nucleotide identity among the circulating G3 strains was 99.52%, while the nucleotide similarity between the current G3 strains and the previously reported G3P[8] Ethiopian strain ranged from 99.3% to 99.5%. Phylogenetic analysis clustered the current strains with classic human RVA G3 strains, distinct from the zoonotic emerging equine-like G3 lineage (Figure 4).

**Figure 5:**
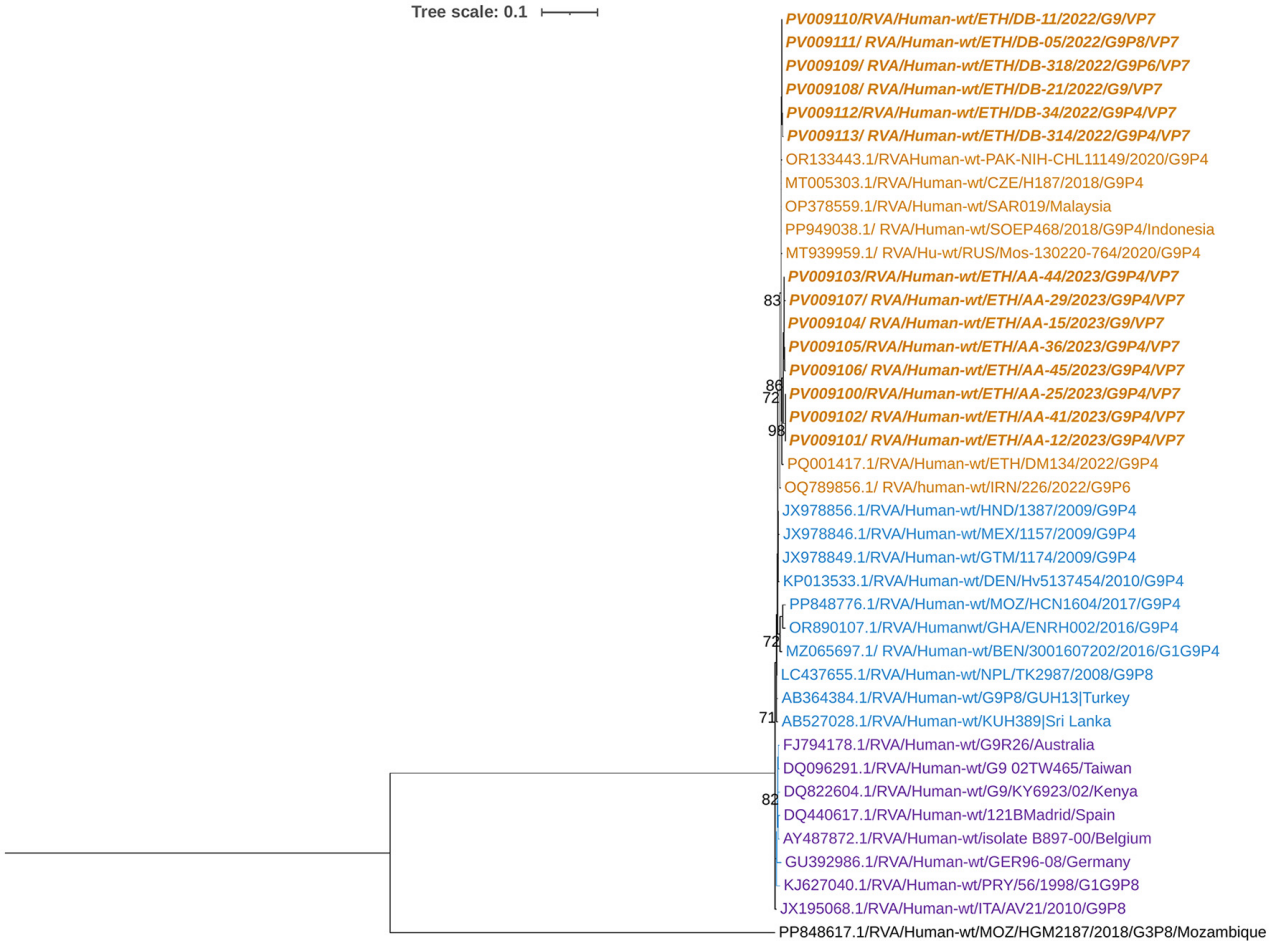
Maximum-likelihood trees of Rotavirus G9 Strains were constructed based on the partial VP7 CDS region sequences (881 base pairs). A T92+G nucleotide substitution model was used to construct the phylogenetic tree. The human RVA G3 strain (PP848617.1) was used as the outgroup. The current Ethiopian strains are written in bold and italic. Each color represents a specific clade. Bootstrap values (1,000 replicates) >70% are shown at the nodes.

The circulating G9 strains showed high sequence identity, ranging from 98.9% to 100% to each other. The majority (8 out of 14) were linked to the P[4] genotype. Phylogenetic analysis placed these strains within the same group as human RVA G9 strains reported from Pakistan, the Czech Republic, Malaysia, Indonesia, Russia, and Ethiopia (Figure 5).

**Figure 6:**
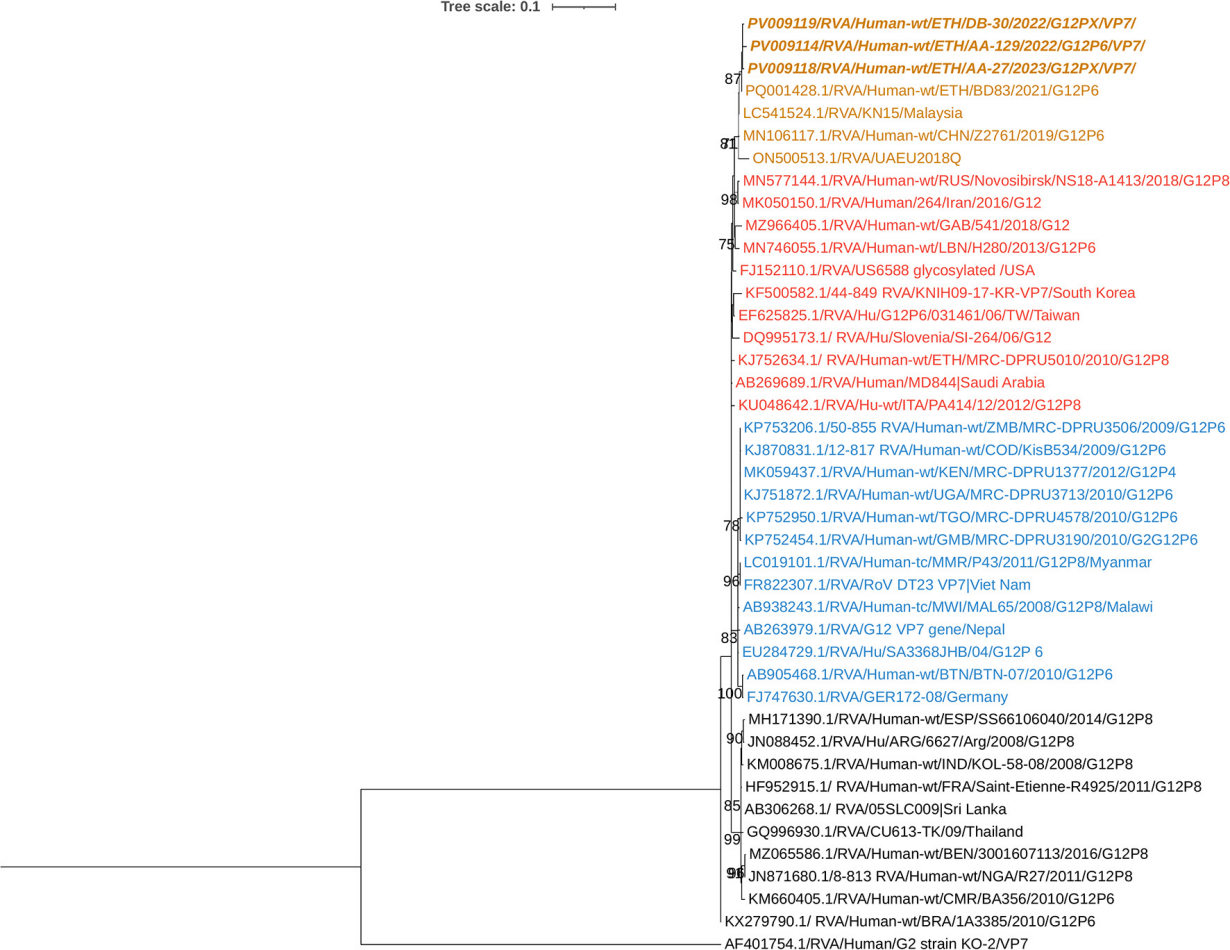
Maximum-likelihood phylogenetic trees of rotavirus G12 strains were constructed using partial VP7 coding region sequences (881 base pairs). The Tamura 3-parameter (T92) model was applied for tree construction. Human RVA G2 strain (AF401754.1) served as the outgroup. Ethiopian strains from this study are indicated in bold and italics. Each color represents a specific clade. Bootstrap values (1,000 replicates) ≥70% are shown at the nodes.

The G12 RVA strains identified in this study showed nucleotide sequence identity ranging from 99.03% to 99.5% among the circulating strains. Phylogenetic analysis clustered these G12 strains with human RVA strains previously reported from Ethiopia, Malaysia, China, the UAE, Japan, and other countries (Figure 6).

### Phylogenetic analysis of the VP4 gene of the circulating RVA strains

**Figure 7:**
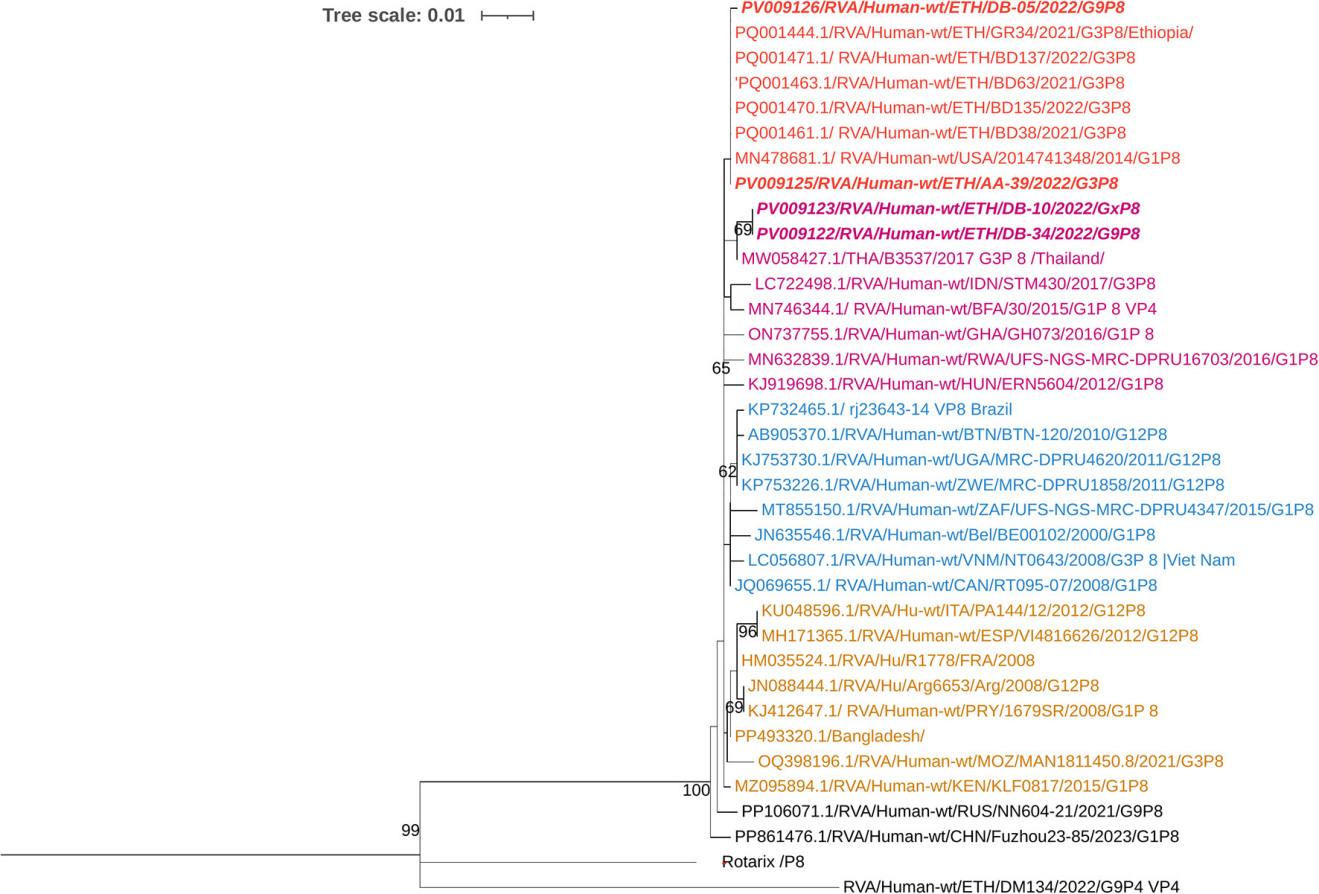
Maximum-likelihood phylogenetic trees of rotavirus P[8] strains were constructed using partial VP4 coding region sequences (877 base pairs). The Tamura 3-parameter with invariant sites (T92+I) model was applied for tree construction. The Human RVA P[4] strain (RVA/Human-wt/ETH/DM134/2022/G9P4) was used as the outgroup. Ethiopian strains from this study are indicated in bold and italics. Each color represents a specific clade. Bootstrap values (1,000 replicates) ≥70% are shown at the nodes.

Phylogenetic analysis revealed that the current circulating P[8] RVAs were closely related to strains previously reported from Ethiopia, India, the USA, and Thailand. In contrast, the Rotarix^®^ P[8] strain was placed in a separate group (Figure 7). The nucleotide sequence identity among the current circulating P[8] RVAs ranged from 99.6% to 100%, whereas their similarity with the Rotarix^®^ P[8] strain varied from 90.6% to 91%.

**Figure 8:**
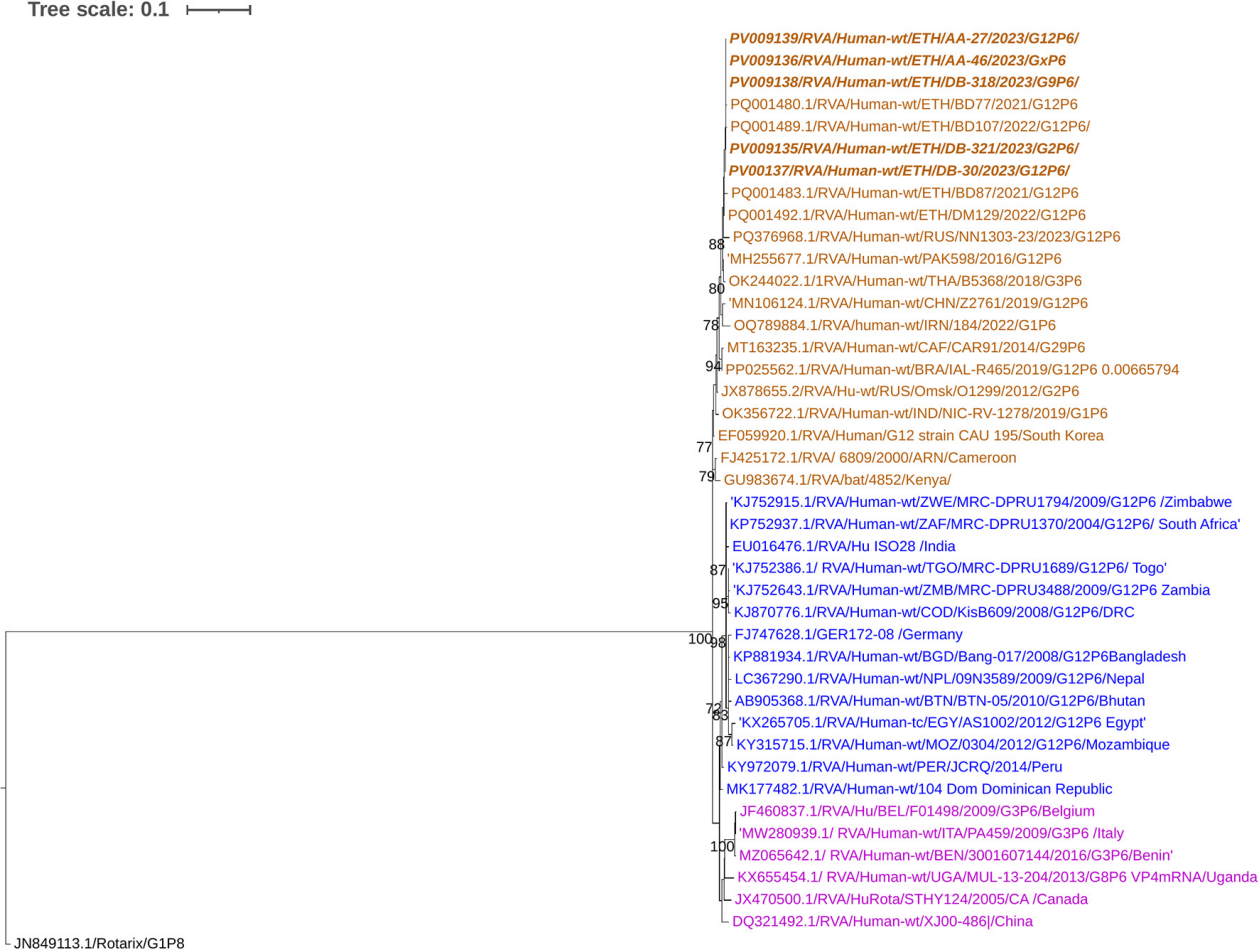
Phylogenetic analysis of rotavirus P [6] strains. A maximum-likelihood tree was constructed based on partial VP4 gene sequences (877 base pairs) using the Hasegawa-Kishino-Yano model with invariant sites (HKY + I). The Human RVA P [8] Rotarix^®^ strain (JN849113.1) was used as the outgroup. P [6] Ethiopian strains from this study are indicated in bold and italics. Each color represents a specific clade. Bootstrap values (1,000 replicates) ≥70% are shown at the nodes.

The circulating P[6] RVAs demonstrated a high nucleotide identity among themselves, ranging from 99.87% to 100%. Compared to previously reported P[6] strains from Ethiopia, they showed a nucleotide identity of 99.1% to 99.8%. The currently circulating P[6] RVAs strain clustered with human RVA strains isolated from children in Ethiopia, Pakistan, Russia, Thailand, China, Iran, the Central African Republic (CAF), Brazil, and India (Figure 8).

**Figure 9:**
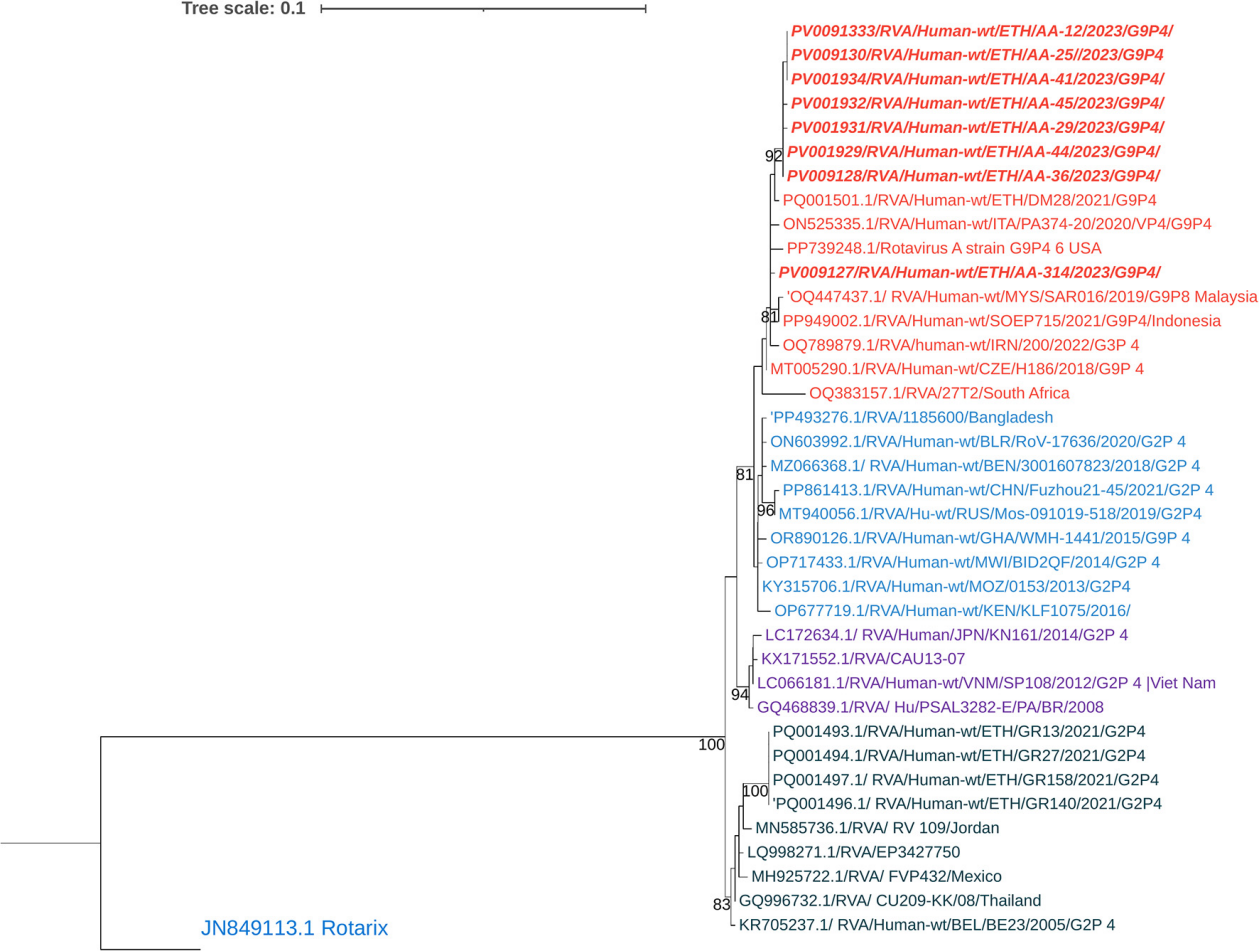
Maximum-likelihood phylogenetic trees of Rotavirus P [4] strains were constructed using partial VP4 coding region sequences (877 base pairs). The Tamura 3-parameter with invariant sites (T92 + I) model was applied for tree construction. The Rotarix^®^ strain P [8] (JN849113.1) was used as the outgroup. Ethiopian strains from this study are indicated in bold and italics. Each color represents a specific clade. Bootstrap values (1,000 replicates) ≥70% are shown at the nodes.

Phylogenetic analysis of the P[4] sequences was conducted for eight RVA strains circulating in the study area. All P[4] strains were associated with G9 and identified from the same area, Addis Ababa. The nucleotide sequence identity among the circulating P[4] strains ranged from 99.4% to 100%. The current P[4] strains clustered closely with wild-type G9P[4] human RVA strains reported from Ethiopia, Italy, the USA, Pakistan, Indonesia, Iran, the Czech Republic, and South Africa. In contrast, previously reported Ethiopian P[4] strains associated with G2 were grouped in separate clusters (Figure 9).

### Comparison of the VP7 and VP4 antigenic epitopes with vaccine strains

**Figure 10:**
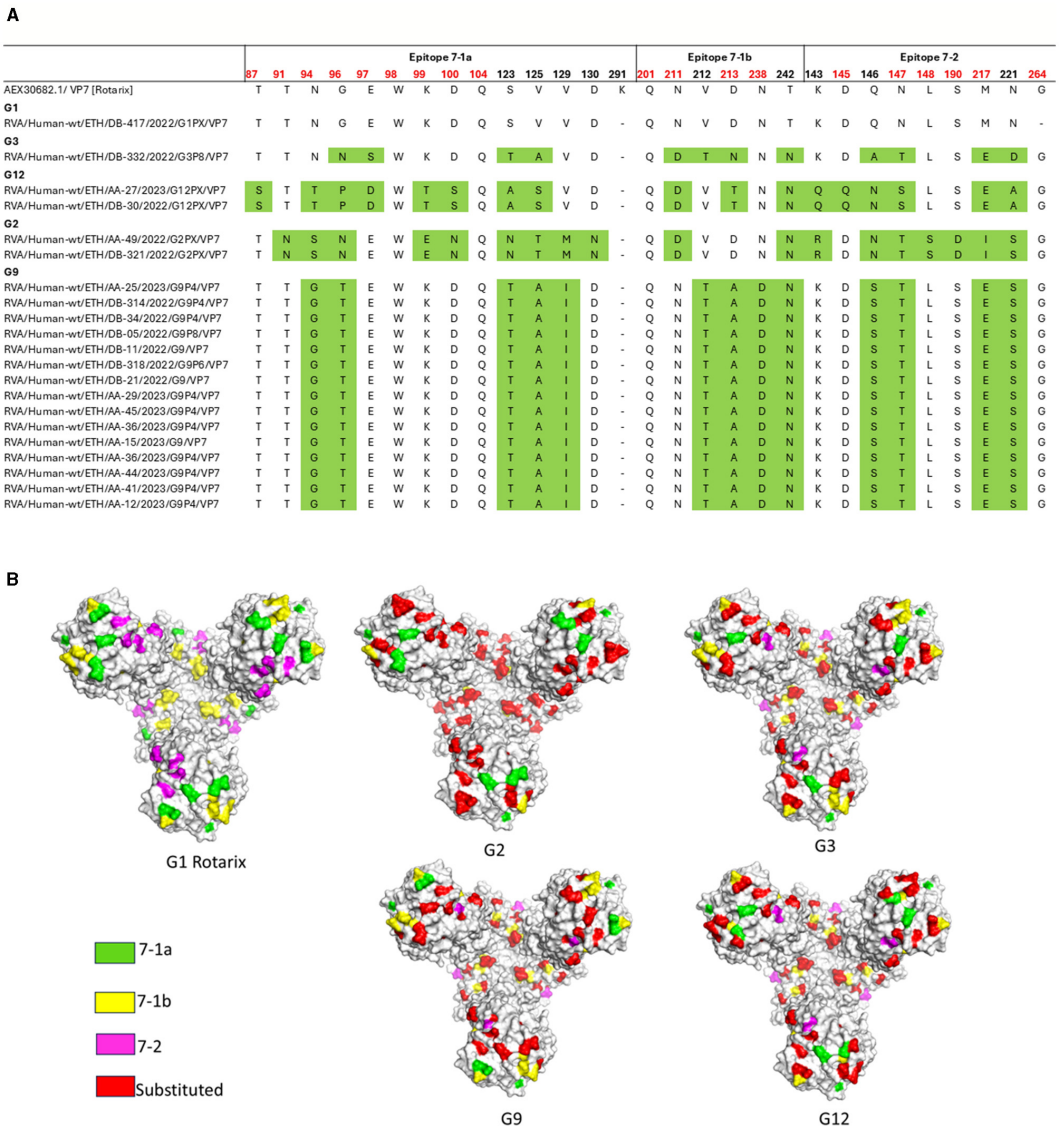
Antigenic variability in the VP7 protein of RVA strains circulating in Ethiopia compared to the Rotarix^®^ vaccine strain. **(A)** Alignment of amino acid residues within antigenic epitopes of Ethiopian RVA VP7 sequences compared with the Rotarix^®^ vaccine strain. Differences are highlighted in green; residues linked to neutralization escape are shown in red. **(B)** Surface representation of the VP7 trimer (PDB ID: 3FMG), illustrating amino acid substitutions identified in Ethiopian RVA strains. Antigenic epitopes 7-1a, 7-1b, and 7-2 are colored green, yellow, and pink, respectively, while surface-exposed substitutions relative to the Rotarix^®^ strain are highlighted in red. Structure rendered using PyMOL (Schrödinger, LLC).

Comparative analysis of the major antigenic epitopes (7-1a, 7-1b, and 7-2) on the VP7 glycoprotein revealed notable sequence divergence between the currently circulating RVA strains and the Rotarix^®^ G1P[8] vaccine strain. The circulating G1 strain (*n* = 1) demonstrated 100% amino acid identity across all epitope regions. In contrast, the G2, G3, G9, and G12 strains exhibited 18, 12, 13, and 17 amino acids substitutions, respectively, across the 29 epitope-defining positions (Figures 10A, B).

**Figure 11:**
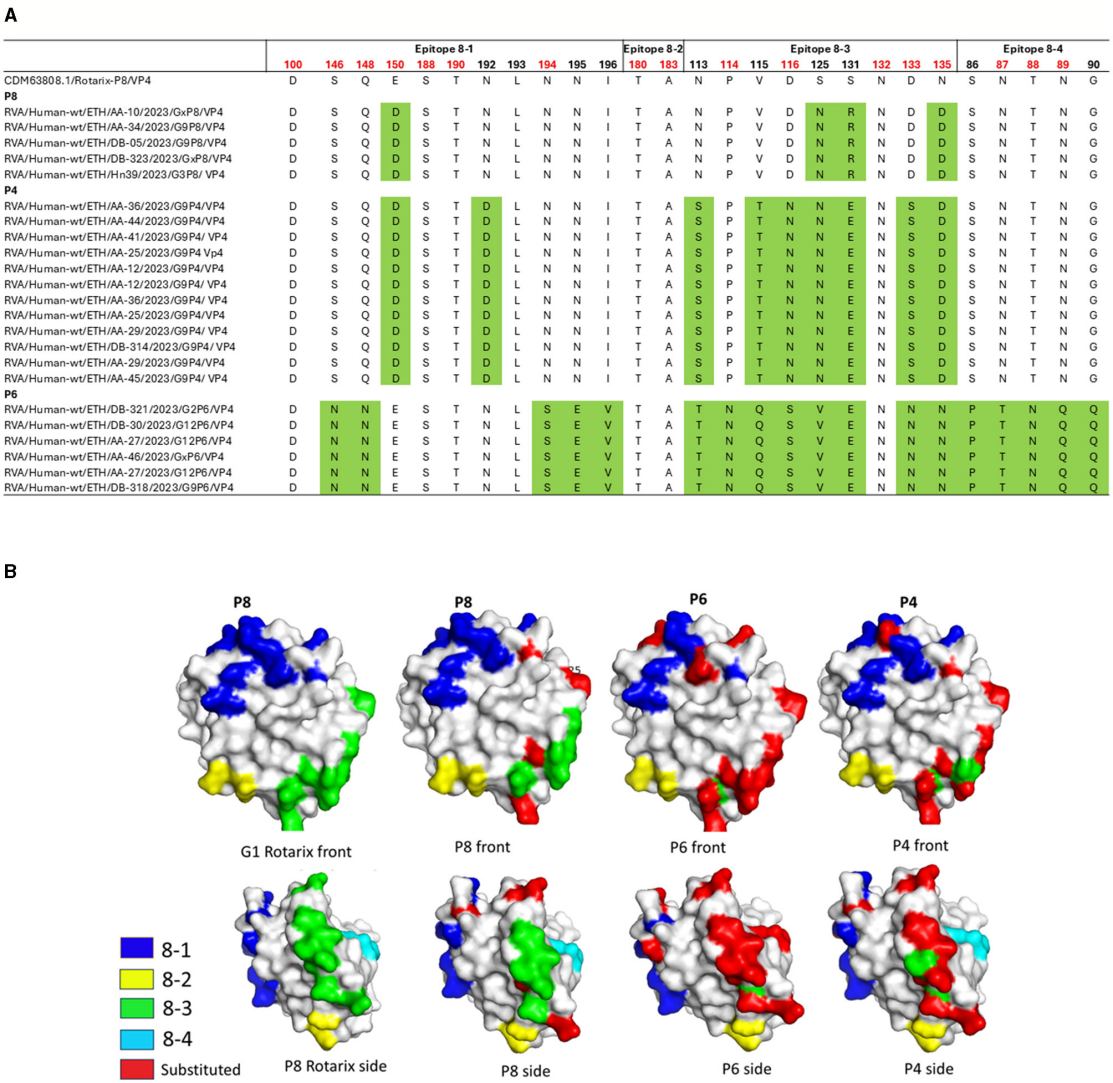
Antigenic variability in the VP8* domain of the VP4 protein in rotavirus RVA strains circulating in Ethiopia compared to the Rotarix^®^ vaccine strain. **(A)** Alignment of amino acid residues within defined antigenic epitopes of the VP8* domain, comparing Ethiopian RVA sequences with the Rotarix^®^ vaccine strain. Amino acid differences are highlighted in green; residues associated with neutralization escape are indicated in red. **(B)** Surface representation of the P[8] monomer (PDB ID: 1KQR). The upper and lower panels show the front and rear views of the VP8* structure. Antigenic epitopes 8-1 through 8-4 are colored blue, yellow, green, and cyan, respectively. Surface-exposed substitutions relative to the vaccine strain are marked in red. Visualization and annotation were performed using PyMOL (Schrödinger, LLC).

The analysis of the amino acid sequences of the four major neutralizing epitopes (8-1, 8-2, 8-3, and 8-4) within the VP8^*^ domain of the VP4 protein across circulating RVA strains compared to the Rotarix^®^ vaccine strains revealed that all five circulating P[8] strains showed four amino acid differences compared to the Rotarix^®^ P[8] strain. Comparative analysis further revealed that P[4] and P[6] strains showed greater divergence from the Rotarix^®^ P[8] strain, with amino acid differences at 9 and 18 of the 28 key epitope residues, respectively. Substitutions such as E150D and N135D in P[8]; E150D, D116N, D133S, and N135D in P[4]; and S146N, Q148N, N149S, P114N, D116S, D133N, N135D, N87T, T88N, and N89Q in P[6] occurred at residues associated with neutralization escape. Among these, N135D (in both P[8] and P[4]), D116S and D133N (P[4]), and P114N and S125V (P[6]) represent non-conservative substitutions within key neutralizing epitopes. While epitope 8-2 remained conserved among all P[4], P[6], and P[8] strains, the highest level of variation was observed in P[6] strains, particularly within epitopes 8-3 and 8-4 (Figures 11A, B).

## Discussion

In this study, the occurrence of rotavirus infection among children with diarrhea was 12.14%, which is lower than the rates reported in earlier studies from Ethiopia (Abebe et al., 2018, 2014; Aliyo, 2022; Damtie et al., 2020; Gelaw et al., 2018; Mwenda et al., 2010; Yassin et al., 2012). This decrease may be attributed to differences in regional socio-economic and environmental factors, the positive impact of rotavirus vaccination programs (Tosisa et al., 2024), and improved hygiene and health practices following the COVID-19 pandemic, as observed in other countries (Nas and Gözüküçük, 2024).

The reported 12.14% is notably lower than rates reported in several other countries, including Kenya (14.5%) (Muendo et al., 2018), Somalia (33.5%) (Roble et al., 2024), and Sri Lanka (36.5%) (Palihawadana et al., 2018). It is also below the pooled estimates for Sub-Saharan Africa (19.95%) and South Asia (17.3%) (Sobi et al., 2024). These variations could be influenced by several factors, such as differences in rotavirus vaccine coverage, diagnostic methodologies, healthcare system capacity, and the genetic diversity of circulating rotavirus strains in each region.

Additionally, the highest infection rate (14.8%) was found in children aged 13–36 months, suggesting a potential age-related susceptibility to rotavirus. This period is particularly vulnerable as it corresponds with the decline of passive maternal immunity and an increase in social and environmental exposure (Lykouretzos and Reiss, 2022).

Our study identified five distinct G-types among the circulating RVA strains: G1, G2, G3, G9, and G12. Notably, G9 emerged as the predominant G-type, accounting for 50% of the detected strains. This finding contrasts with previous Ethiopian studies, which consistently reported G3 as the dominant genotype, followed by G12 and G1 (Damtie et al., 2020; Tosisa et al., 2024). This shift suggests a major genotype replacement event, likely driven by selective pressure from the Rotarix^®^ vaccine. Our result aligns with previous studies that have reported the global dominance of G9 in recent years (Dong et al., 2023; Kiulia et al., 2014; Le et al., 2024; Matthijnssens et al., 2010). The high prevalence of G9 could be attributed to various factors, including enhanced transmissibility, immune evasion mechanisms, or a combination of both (Dong et al., 2023). Further analysis of the circulating G9 strains revealed high sequence identity, ranging from 98.9% to 100%. These strains clustered within the same phylogenetic group as G9 strains previously reported from Ethiopia, suggesting a potential common origin or recent transmission events.

G12 (10.7%) and G2 (7.1%) were the second and third most common G-types, respectively. The presence of these genotypes highlights their importance in the current epidemiology of rotavirus infections. G12 has been increasingly reported worldwide, especially in Africa and India, where it shows a notable prevalence among hospitalized children (Arun et al., 2019; Mokoena et al., 2021). Although less common, G2 continues to play a significant role in rotavirus infections due to its involvement in genetic diversity and reassortment events (Jere et al., 2011; Miao et al., 2025). G2 rotavirus causes severe gastroenteritis, particularly in infants and young children, and has been linked to major outbreaks (Lugonja et al., 2020; Stojkovska et al., 2020; Tate et al., 2016). It may also cause extra-intestinal symptoms, such as neurological and respiratory complications (Dian et al., 2021).

The low occurrence of G1 rotavirus strains in the current study contrasts with previous studies, indicating their prevalence in the country (Abebe et al., 2018; Aliyo, 2022; Damtie et al., 2020; Tosisa et al., 2024). This decline may reflect shifts in viral ecology or the influence of vaccination programs, which could exert selective pressure on these strains.

Further genetic analysis of the current G1 strain revealed that its viral sequence shared a high nucleotide identity (96.34%) with the G1 strain in the Rotarix^®^ vaccine. This high genetic similarity suggests that the vaccine may still confer immunity against the circulating G1 strain.

Among the rotavirus P-types identified, P[4] was the most prevalent (28.6%), followed by P[6] (21.4%) and P[8] (17.9%). This pattern differs from earlier studies in Ethiopia, where P[8] was the predominant type (Abebe et al., 2018, 2014; Aliyo, 2022; Damtie et al., 2020; Gelaw et al., 2018; Mwenda et al., 2010; Yassin et al., 2012). These findings indicate that rotavirus types can evolve, emphasizing the importance of regular monitoring to guide vaccine planning and safeguard public health.

Circulating P[8] rotavirus strains showed a notably low genetic similarity of 90.6% to 91% to the Rotarix^®^ P[8] vaccine strain. This finding was confirmed by phylogenetic analysis, which showed that the circulating strains clustered separately from the vaccine strain. This significant genetic distance may have important implications for vaccine effectiveness.

Genetic analysis of the circulating P[4] rotavirus strains in the current study revealed a high level of nucleotide sequence similarity, ranging from 99.4% to 100%. All P4 strains were associated with the G9 genotype and were identified exclusively from Addis Ababa. This finding points to a highly clonal and likely localized circulation of a specific G9P[4] strain within Addis Ababa during the study period. Therefore, the identification of a clonal G9P[4] rotavirus population highlights the potential to cause localized outbreaks and underscores the critical need for continuous, robust molecular surveillance. Phylogenetic analysis showed that these strains clustered with G9P[4] human RVA strains previously reported from Ethiopia and other countries, including Pakistan, the Czech Republic, Malaysia, Indonesia, and Russia. The close genetic relationship indicates that the current clones may have evolved from earlier circulating strains in Ethiopia.

G9P[4] was the dominant G/P genotype combination in the current study, accounting for 35% cases. Its predominance suggests its strong adaptability and potential fitness advantage, making it an important candidate for consideration in future vaccine strategies. G9P[4] has increasingly been reported as a significant strain associated with acute gastroenteritis in children, indicating a possible shift in global rotavirus epidemiology. Its emergence and dominance have been documented in several countries, including Iran (Kachooei et al., 2023), Mexico (Felix-Valenzuela et al., 2016), Pakistan (Usman et al., 2024), and Guatemala (Quaye et al., 2013), emphasizing its global relevance and the need for continued monitoring.

Emerging rotavirus strains such as G12P[6] and G9P[8], though less common, are gaining attention due to their increasing prevalence and potential public health impact. In Africa, these genotypes have spread across several countries following the introduction of rotavirus vaccines (Rakau et al., 2021). In Turkey, G12P[6] accounted for 11% of pediatric gastroenteritis cases, indicating its growing significance (Aydin and Aktaş, 2017). Whole-genome analyses suggest that G12P[6] may have undergone reassortment with porcine strains, reflecting its genetic adaptability (Mokoena et al., 2021). Similarly, G9P[8] has become a common strain after vaccine introduction among the general public (Abebe et al., 2014; Aliyo, 2022; Gelaw et al., 2018; Tosisa et al., 2024). This genotype has been widely reported in different countries, including Tunisia (Bennour et al., 2020), Malaysia (Fong et al., 2024; Tahar et al., 2023), China (Jiao et al., 2023), and Japan (Kawata et al., 2021), underscoring its global relevance.

The G3P[8] rotavirus strain was detected at a low prevalence (4%) in the current study, compared with previous reports from Ethiopia (Aliyo, 2022; Damtie et al., 2020; Tosisa et al., 2024). The genetic analysis revealed that the circulating G3 strains are closely related to previously reported G3P[8] Ethiopian strains, with a nucleotide identity ranging from 99.3% to 99.5%. The high genetic similarity among these strains confirms their close evolutionary relationship. Phylogenetic analysis showed that the current isolates clustered with classical human RVA G3 strains and were distinct from the emerging equine-like G3 lineage. These findings suggest that the circulating G3 strains in this study are of human rather than zoonotic origin.

The observation that 25% and 32.14% of samples were deemed untypeable for P-type and G-type, respectively, highlights significant challenges in rotavirus genotyping. The difficulty in accurately typing rotavirus samples might be due to high genetic variability and the presence of rare genotypes, which complicate the genotyping process (Adah et al., 2003; Dellis et al., 2024). Mutations at primer binding sites and mixed infections further hinder sequencing accuracy (Iturriza-Gómara et al., 2001). Due to high mutation rates, 28.3% of rotavirus samples were untyped from primer mismatches (Mitui et al., 2012). In Brazil, 86.9% of initially untypeable cases could later be classified into genotypic combinations (Willame et al., 2018). These issues have important implications for public health surveillance, as a high proportion of untypeable strains may indicate the presence of novel or uncommon variants that are not accounted for in current monitoring systems or vaccine formulations. This underscores the need for improved primer design and the adoption of Next-Generation Sequencing (NGS) approaches to enhance genotyping resolution and ensure comprehensive strain characterization.

A comparative analysis of VP7 and VP4 antigenic epitopes revealed substantial amino acid variability between circulating Ethiopian RVA strains and the Rotarix^®^. Multiple mutations were detected within neutralizing epitopes across various genotypes, which may have implications for vaccine effectiveness. Similar antigenic divergence has been observed in China (Mao et al., 2022), Qatar (Mathew et al., 2023), Belgium (Zeller et al., 2012), and Gabon (Manouana et al., 2021), emphasizing the need for continued monitoring of rotavirus strain evolution.

The comparison between the circulating G1 rotavirus and the Rotarix^®^ vaccine strain revealed no amino acid substitutions within the VP7 antigenic epitopes, suggesting a high degree of genetic conservation. On the other hand, G2, G3, G9, and G12 strains exhibited significantly higher amino acid variability in VP7 antigenic epitopes, with 18, 12, 13, and 17 substitutions, respectively, compared to the Rotarix^®^ vaccine G1 strain. This suggests potential limitations in vaccine-induced immunity against heterotypic (non-G1) strains (Esona et al., 2011; Fallah et al., 2024; Zeller et al., 2012). These substitutions, linked to neutralization escape, have been observed in studies from the USA and other regions (Esona et al., 2011; Motamedi-Rad et al., 2020), indicating that such variability may reduce vaccine effectiveness.

Circulating P[8], P[4], and P[6] RVA strains exhibited substantial amino acid substitutions in the VP8^*^ region of VP4 neutralizing epitopes compared to Rotarix^®^. Non-conservative substitutions occurred at residues associated with neutralization escape, including N135D, present in both P[8] and P[4], which introduces a negative charge; D116S and D133N in P[4], which remove negative charges and could disrupt electrostatic interactions; P114N, which replaces a rigid proline with asparagine, potentially altering local backbone stability; and S125V in P[6], which changes a polar residue to hydrophobic, possibly reducing epitope accessibility (Kazimbaya et al., 2018). These changes may undermine vaccine effectiveness against both homotypic and heterotypic responses (Motamedi-Rad et al., 2020; Xu et al., 2019) and could help explain the continued high prevalence of RVA infection despite high immunization coverage in the country.

Although the pentavalent RotaTeq^®^ and Rotasiil^®^ vaccines are not part of Ethiopia's national immunization program, their broader formulation theoretically offers wider coverage than Rotarix^®^ However, its potential superiority against the strains circulating in Ethiopia remains uncertain. Numerous studies from diverse global settings have shown that RotaTeq^®^ also faces challenges from antigenic drift, with significant amino acid mismatches and neutralization escape mutations reported between its vaccine components and wild-type strains (Manouana et al., 2021; Mao et al., 2022; Mathew et al., 2023; Ogden et al., 2019; Zeller et al., 2012). Therefore, it is unclear if RotaTeq^®^ would provide substantially better protection in the Ethiopian context. This highlights a critical gap and reinforces the urgent need for next-generation multivalent vaccines tailored to the specific genotypes prevalent in high-burden regions like sub-Saharan Africa. Multivalent vaccines such as Rotasiil^®^ represent promising alternatives, as they broaden strain coverage and offer additional advantages in efficacy, safety, and thermostability (Kanungo et al., 2022), making it well-suited for rotavirus control in resource-limited settings.

## Conclusion

This study highlights the genetic diversity and evolving nature of circulating rotavirus strains in the region. The predominance of G9P[4] and the detection of multiple amino acid substitutions in both VP7 and VP4 antigenic regions, particularly in non-G1 genotypes, suggest potential challenges to current vaccine-induced immunity. The observed divergence from the Rotarix^®^ vaccine strains, especially in key neutralizing epitopes, raises concerns about reduced vaccine effectiveness against heterotypic strains. Furthermore, the close phylogenetic clustering with strains from diverse global regions underscores the interconnectedness of rotavirus epidemiology. These findings emphasize the need for continuous molecular surveillance and consideration of genotype diversity in future vaccine development and immunization strategies.

## Data Availability

The datasets presented in this study can be found in online repositories. The names of the repository/repositories and accession number(s) can be found in the article/supplementary material.
